# Transport Characteristics of Branched-Preformed Particle Gel in Porous Media: Influence of Elastic Modulus, Matching Coefficient, and Injection Rate

**DOI:** 10.3390/gels11050315

**Published:** 2025-04-23

**Authors:** Ruping Chen, Hong He, Yuhang Tian, Zixiang Xiong, Wenli Ke, Haihua Pei, Peng Zhang

**Affiliations:** 1 College of Petroleum Engineering, Yangtze University, Wuhan 430100, China; 15071198187@163.com (R.C.); 18064292212@163.com (Y.T.); 16608647005@163.com (Z.X.); kewenli2006@163.com (W.K.); 2 Key Laboratory of Drilling and Production Engineering for Oil and Gas, Wuhan 430100, China; 3 School of Petroleum Engineering, China University of Petroleum (East China), Qingdao 266580, China; peihaihua@upc.edu.cn; 4 School of Chemistry and Chemical Engineering, Chongqing University of Science & Technology, Chongqing 401331, China; zhangpeng@cqust.edu.cn

**Keywords:** transport characteristics, branched-preformed particle gel, elastic modulus, matching coefficient, porous media

## Abstract

The viscoelastic branched-preformed particle gel (B-PPG) has been successfully applied to enhance oil recovery in mature reservoirs. However, due to a lack of a clear understanding of the transport characteristics of B-PPG in porous media, the injectivity and plugging efficiency are not ideal, and the incremental oil recovery is not as expected, which poses a great obstacle to the large-scale popularization and application of B-PPG in mature oilfields. Thus, the influences of elastic moduli, matching coefficients, and injection rates on transport characteristics of B-PPG in porous media were investigated by conducting core flow experiments. The results indicate that the elastic modulus of B-PPG can significantly affect the injectivity and plugging efficiency. The higher the elastic modulus is, the more difficult it is to transport in the porous medium. When the particle size is similar, as the elastic modulus increases, the resistance factor (*F_r_*) and residual resistance factor (*F_rr_*) increase. When the elastic modulus is similar, as the particle size increases, the *F_r_* and *F_rr_* increase. As the matching coefficient decreases, the *F_r_* and *F_rr_* decrease, reflecting the improvement of injectivity and the weakening of plugging efficiency. The higher the reservoir permeability, the lower the matching coefficient. When the reservoir permeability ranges from 0.30 to 5.30 μm^2^, the B-PPG with an elastic modulus of 42.2 Pa and a *D*_50_ of 525 μm can migrate smoothly into the depth of porous media and form effective plugging. As the injection rate increases, the *F_r_* and *F_rr_* decrease, reflecting the improvement of injectivity and the weakening of plugging efficiency. Therefore, to achieve good injectivity and plugging efficiency of the B-PPG suspension, the injection rate should be in the range of 0.5 mL·min^−1^ to 1.5 mL·min^−1^. Hence, these findings could give an important understanding of the factors affecting the transport characteristics of B-PPG and provide guidance for enhancing oil recovery by B-PPG flooding in mature oilfields.

## 1. Introduction

Water flooding is commonly used to maintain formation energy and improve oil recovery. However, as the waterflooded reservoirs enter into the development stage with high water-cut, the heterogeneity of the reservoir becomes serious, and preferential water flow paths are formed in the reservoir. The injected water flows along the preferential channel and results in low sweep efficiency. It is predicted that around 60~75% of the remaining oil still exists in the unswept zone after water flooding [1,2,3,4,5]. Therefore, it is crucial to recover the unswept remaining oil to further enhance oil recovery. Many enhanced oil recovery (EOR) techniques have been proposed to improve sweep efficiency and oil displacement efficiency, including conformance control or water shutoff technology, chemical flooding [6,7,8,9,10,11,12], gas flooding [13,14,15,16,17], foam flooding [18,19,20], and so on.

Conformance control or water shutoff technology can achieve the EOR goal by plugging preferential water flow channels and diverting the subsequent water into the unswept area to improve the sweep efficiency [21,22,23,24]. Polymer gels are most commonly used for water shutoff or conformance control treatments in mature waterflooded reservoirs. Polymer gels refer to using polyacrylamide and a crosslinker to form a three-dimensional network structure under reservoir conditions [25,26,27]. Nevertheless, due to the effects of shear, adsorption, and dilution, there exists uncertainty in gelation for polymer gels under reservoir conditions. To address this problem, preformed particle gel (PPG) has been extensively studied [28,29,30,31]. Different types of PPG are synthesized by acrylamide monomer, crosslinker, initiator, and additives under surface conditions. After swelling, PPG has elasticity and strength, which can pass through the pore throat by deformation under pressure [32,33,34]. PPG shows promising potential for conformance control in mature waterflooded reservoirs.

Polymer flooding, a common chemical flooding technique, has been successfully applied for EOR by reducing the mobility ratio of water and oil and expanding sweep efficiency. However, due to greater reservoir heterogeneity and more dispersed remaining oil, there still exists remaining oil that is unrecovered after polymer flooding. In addition, the EOR efficiency of subsequent surfactant-polymer (SP) flooding after polymer flooding is low [35]. Therefore, based on the SP flooding technology, the concept of heterogeneous phase combined flooding (HPCF) technology was proposed. The HPCF system consists of polymer, branched-preformed particle gel (B-PPG), and surfactant, which can achieve higher EOR efficiency through the synergistic effect. Compared with traditional PPG, B-PPG has better suspension capacity and viscoelastic properties [36,37]. The viscoelastic B-PPG has been successfully used in the HPCF system for EOR in mature reservoirs after polymer flooding. However, due to the lack of a clear understanding of the transport characteristics of B-PPG in reservoirs, the injectivity and plugging efficiency are not ideal, and the incremental oil recovery is not as expected, which poses a great obstacle to the large-scale popularization and application of B-PPG in mature oilfields. Thus, it is urgent to investigate the transport characteristics of B-PPG suspension to better understand the injectivity and plugging performance [38,39].

Extensive studies have been conducted to clarify B-PPG transport behavior. Zhao et al. [40] investigated the transport mechanisms of B-PPG in porous media at the microscopic scale. The results showed that there were five ways for B-PPG to pass through the pore throat: unrestricted transport, deformational transport, fragmentation-induced transport, adsorption–retention, and pore-throat plugging. Li et al. [41] found that B-PPG exhibited better stability and long-term anti-aging properties under high temperature and salinity conditions. Moreover, the migration of B-PPG particles in the porous medium is a dynamic process of plugging and passing through. Liu et al. [42] explored the adaptability to different permeabilities and the plugging characteristics of B-PPG. The results showed that B-PPG could form temporary and repetitive single-particle or multi-particle accumulations. When the permeability was in the range of 1.6 to 3.2 μm^2^, B-PPG could migrate to the deep reservoir, significantly improving conformance in heterogeneous reservoirs. Gong et al. [43] evaluated the seepage characteristics of B-PPG/HPAM using the resistance coefficient and residual resistance coefficient as evaluation indicators. The combination of B-PPG with polymers could significantly enhance the carrying capacity of the system, and it had a good profile control effect on interlayer heterogeneous reservoirs. Liu et al. [44] investigated the influence of permeability and injection rate on the seepage characteristics of the composite system of B-PPG and polymer in porous media. This system could be smoothly injected into cores with a permeability of 0.5–3.4 μm^2^ and form plugging. When the displacement rate was between 0.5 and 0.75 mL·min^−1^, the system could have both injectability and plugging ability. Tang et al. [45] studied the influence of different permeabilities and injection rates on the injection and plugging effects of B-PPG in fractured cores. When the injection rate was low, B-PPG could migrate to the deep reservoir under high pressure, and B-PPG was suitable for fractured reservoirs with a permeability exceeding 0.1 × 10^−3^ μm^2^. Li et al. [46] found that when the salinity was in the range of 3550 to 10,651 mg·L^−1^, the injectivity of B-PPG improved with the increase of salinity. However, when the salinity exceeded 10,651 mg·L^−1^, the injectivity of the B-PPG dispersion system decreased as the salinity increased. In summary, the ways in which B-PPG passes through the pore throats have been clearly studied and systematically summarized. By means of the radical polymerization method, the internal structure of B-PPG is highly branched and contains a certain amount of three-dimensional network structure. Thus, B-PPG has the deformation ability of traditional PPG and can increase the viscosity of the carrier fluid. However, the above literature survey has mostly focused on exploring factors such as the injection rate and permeability, while the influence of particle mechanical properties on B-PPG seepage characteristics remains poorly understood.

Considering different reservoir permeabilities, injection parameters, and the physical properties of B-PPG, the transport characteristics of B-PPG in reservoirs are complex and still uncertain, which are affected by the elastic modulus, particle size, injection rate, reservoir permeability, and so on. Thus, it is crucial to understand the above influencing factors affecting the transport characteristics of B-PPG in porous media. Therefore, the objective of this research is to investigate the influence of elastic modulus, matching coefficient, and injection rate on the transport characteristics of B-PPG. Firstly, different B-PPG particles with different elastic modulus and particle sizes were evaluated in this study. Then, core flow experiments were performed to investigate the influence of elastic modulus, matching coefficient, and injection rate on the transport characteristics of B-PPG. The study can provide a theoretical basis for the application of B-PPG in enhanced oil recovery within porous media.

## 2. Results and Discussion

### 2.1. Swelling Ratio of B-PPG

**Figure 1:**
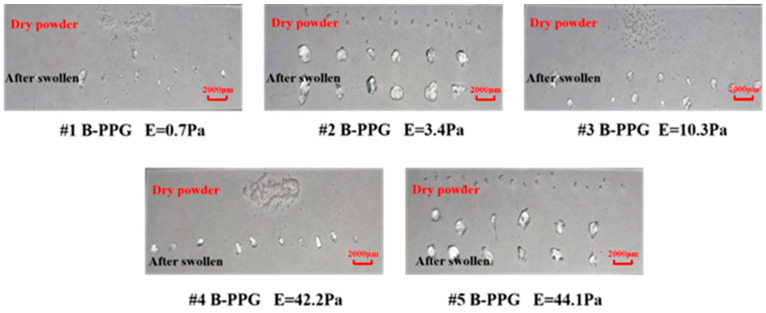
Images of B-PPG before and after swelling.

**Figure 2:**
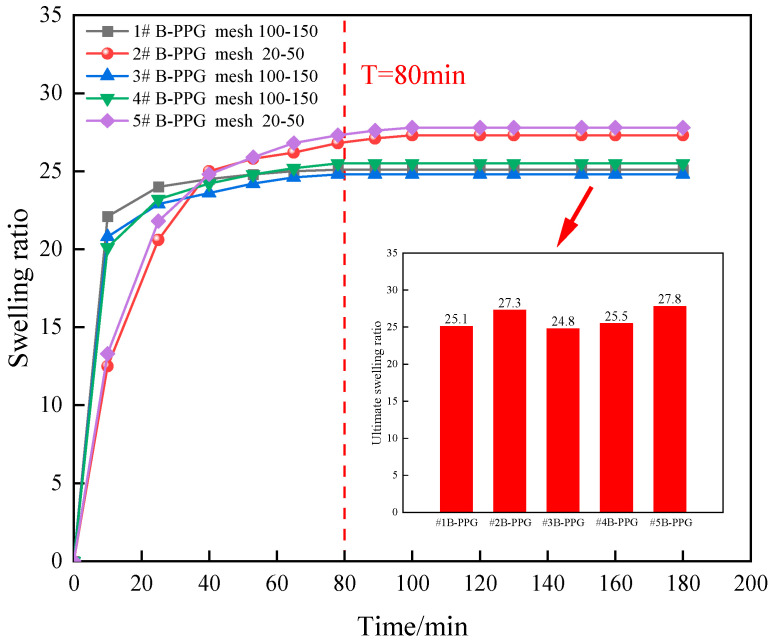
Variation in the swelling ratio of B-PPG samples versus time.

Figure 1 shows images of particles before and after swelling. Figure 2 shows the swelling ratio of B-PPG as a function of time.

The dry size of #1, #3, and #4 B-PPG samples was 100–150 mesh. The dry size of #2 and #5 B-PPG samples was 20–50 mesh. As can be seen from Figure 2, the particles were irregular in shape and had angular edges after swelling. Moreover, the size of #1, #3, and #4 B-PPG samples before and after swelling was clearly smaller than that of #2 and #4 B-PPG samples.

The swelling ratio of B-PPG with different elastic moduli increased rapidly and then decreased to a stable value after 80 min. The initial swelling rate of B-PPG with a smaller size was higher than that of B-PPG with a larger size. The inset figure illustrates the ultimate swelling ratio of five B-PPGs after reaching water absorption equilibrium. The ultimate swelling ratios of #1, #3, and #4 B-PPG samples were in the range of 24.8–25.5, and the ultimate swelling ratios of #2 and #5 B-PPGs were in the range of 27.3–27.8. This indicated that the particle size could affect the swelling rate and have little effect on the ultimate swelling ratio.

### 2.2. Particle Size Distribution and D_50_ Determination of B-PPGs

**Figure 3:**
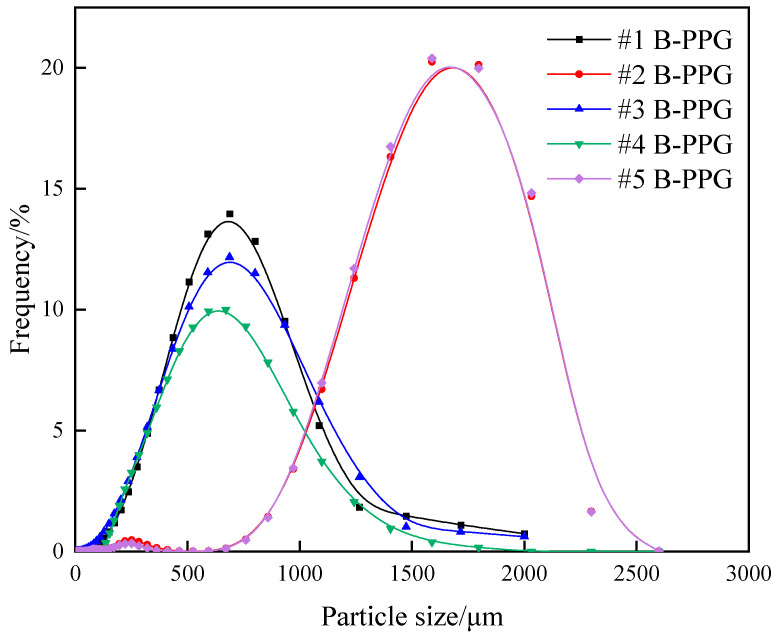
Particle size distribution of B-PPGs with different elastic moduli after swelling.

The ratio of median diameter (*D*_50_) to pore throat diameter (*D_p_*) was defined as the matching coefficient, which is crucial for screening particle gel and its application in the field. Figure 3 shows the particle size distribution of 800 mg·L^−1^ B-PPG with different elastic moduli after swelling.

As can be seen from Figure 3, the particle sizes of #2 B-PPG and #5 B-PPG suspensions are mainly distributed between 1000~2300 μm. The median particle diameter (*D*_50_) represents the particle size at which 50% of the cumulative mass distribution is reached on the particle size distribution curve. The *D*_50_ of #2 B-PPG and #5 B-PPG suspensions can be determined as 1465 μm and 1466 μm, respectively. The particle size distributions of #1 B-PPG, #3 B-PPG, and #4 B-PPG suspensions are mainly distributed between 100~1400 μm, and the *D*_50_ of #1 B-PPG, #3 B-PPG, and #4 B-PPG suspensions can be determined as 550 μm, 542 μm, and 525 μm, respectively. Table 1 summarizes the *D*_50_ of B-PPG suspensions with different elastic moduli. B-PPG is synthesized through crosslinking reactions involving a chemical monomer, crosslinker, and initiator under certain conditions, followed by processing steps including crushing and sieving. The synthesis processes vary among different types of B-PPG. The reasons for the abrupt particle size distribution of #2 and #5 may be attributed to factors such as variations in monomer concentration during synthesis, differences in reaction temperature, as well as changes in agitation mode and stirring speed during the reaction.

### 2.3. FTIR Characterization of B-PPG

**Figure 4:**
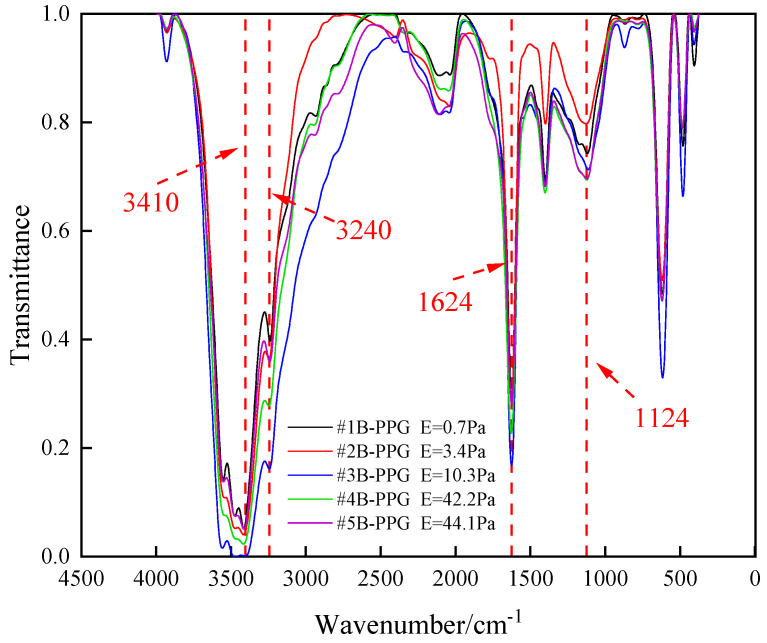
FTIR spectra of B-PPG with different elastic moduli.

FTIR spectroscopy is used to characterize the molecular structure of B-PPG. Figure 4 shows the FTIR spectra of B-PPG with different elastic moduli.

Figure 4 shows the Fourier transform infrared spectrum of B-PPG with different elastic moduli. The broadband absorption peak between 3400 and 3600 cm^−1^ was mainly caused by the -NH_2_ stretching vibration. The absorption peak at 3240 cm^−1^ was attributed to the stretching vibration of -OH. The absorption peak at 1624 cm^−1^ was attributed to C=O stretching vibration of -CONH- or -CONH_2_. The absorption peak at 1124 cm^−1^ was attributed to the stretching vibration of C-N. The FTIR measurement showed that the B-PPG used in this study belongs to polyacrylamide particle gel.

### 2.4. Factors Affecting the Transport Characteristics of B-PPG

#### 2.4.1. Effect of Elastic Modulus of B-PPG

The cross-linked network structure makes the B-PPG elastic and deformable under pressure, and the deformation capacity is characterized by elastic modulus (*E*). It has been demonstrated that the elastic modulus of B-PPG is mainly affected by the composition of the initial bulk gel; the higher the concentration of the main agent and crosslinking agent is, the greater the elastic modulus is. The elastic modulus represents the proportional coefficient between stress and strain in the elastic deformation phase of a material under force. It serves as a critical parameter for quantitatively analyzing the external force required to induce elastic deformation in B-PPG particles. It characterizes the deformability of particles and serves as an effective indicator of whether particles can migrate into deep reservoir formations to form effective plugging. Thus, the elastic modulus is directly related to the deformation capacity of B-PPG under pressure, which is crucial for determining whether it can be injected into porous media smoothly.

In order to clarify the influence of elastic modulus on the transport characteristics of B-PPG, core flow experiments were performed, and pressure drop versus injected pore volume curves, *F_r_*, and *F_rr_* were analyzed and determined. The basic parameters of the sand packs are shown in Table 2.

**Figure 5:**
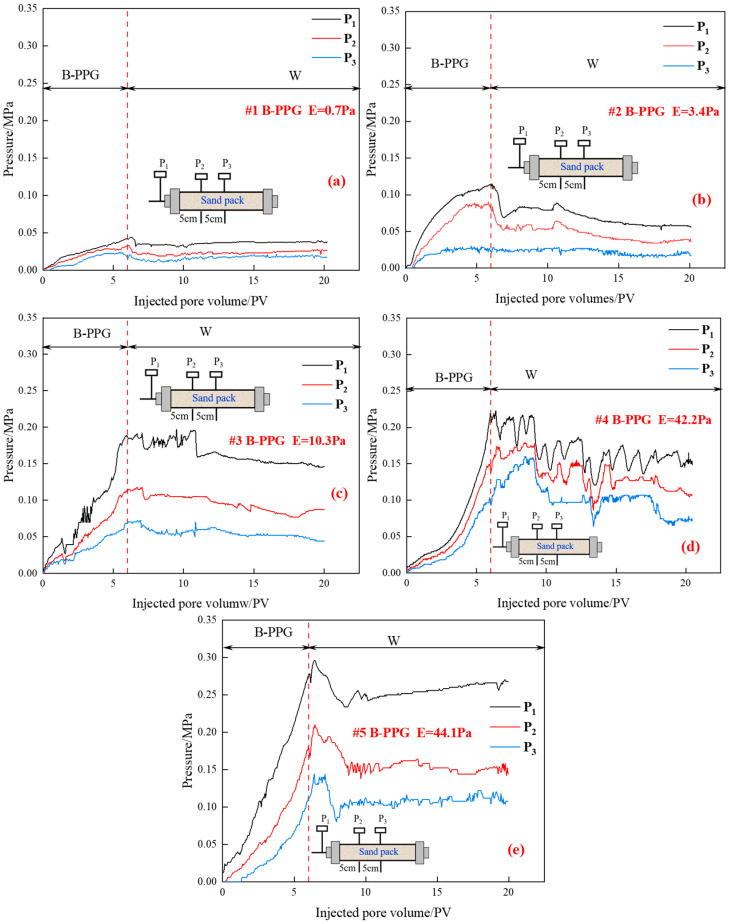
Relationship between the injection pressure and injected pore volumes of different B-PPG systems: (**a**) #1 B-PPG, *E* = 0.7 Pa; (**b**) #2 B-PPG, *E* = 3.4 Pa; (**c**) #3 B-PPG, *E* = 10.3 Pa; (**d**) #4 B-PPG, *E* = 42.2 Pa; (**e**) #5 B-PPG, *E* = 44.1 Pa.

Figure 5 shows the pressure versus injected pore volumes during different flooding periods under different elastic moduli.

As can be seen from Figure 5a,b, it can be found that when the elastic modulus is 0.7 Pa and 3.4 Pa, during the B-PPG suspension injection process, with the increase of injected pore volumes, the injection pressure (P_1_) increases and pressure measuring points P_2_ and P_3_ show the same change trend. As the injected pore volumes increase, the slope of the injection pressure curve decreases, which indicates that regardless of particle size, #1B-PPG and #2B-PPG with low elastic moduli can be injected and transported smoothly in porous media. During the subsequent waterflooding, the injection pressure decreases gradually and reaches a stable level.

However, as can be seen from Figure 5c–e, with the increase of elastic modulus, the slope of the injection pressure curve increases with the increase of injected pore volumes, which indicates that the higher the elastic modulus, the worse the injectivity. During the subsequent waterflooding, an obvious fluctuation of injection pressure can be observed, and it shows a decreasing trend.

Moreover, as can be seen from Figure 5d,e, when the elastic modulus of B-PPG suspension is similar, during the injection process of B-PPG suspension, the injection pressure rising speed increases with the increase of particle size. During the subsequent water flooding process, the pressure curve presents a fluctuating characteristic. For the #4 B-PPG suspension with a smaller size, the fluctuation of injection pressure can be observed and shows a decreasing trend, which indicates that the B-PPG particle can flow in the sand-pack by “migration, plugging, deformation, passing, and remigration”. In contrast, for the #5 B-PPG suspension with the larger size, with the increase of pore volume, the injection pressure fluctuates first and then increases slightly, which indicates that the B-PPG with a larger size plugs the pore throat and has worse migration ability.

**Figure 6:**
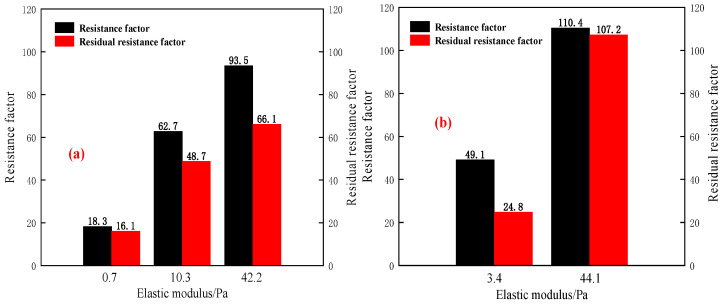
The resistance factors and residual resistance factors versus elastic moduli: (**a**) the median diameter of B-PPG ranges from 525 to 550 μm; (**b**) the median diameter of B-PPG ranges from 1465 to 1466 μm.

In order to further clarify the injectivity and plugging efficiency, the *F_r_* and *F_rr_* are calculated and shown in Table 3 and Figure 6.

As can be seen from Figure 6a,b, with the increase of the elastic modulus of B-PPG, the *F_r_* and *F_rr_* increase. The lower the elastic modulus, the stronger the deformation capacity of B-PPG under pressure. On the one hand, the B-PPG with a low elastic modulus can be injected into the porous media more easily. On the other hand, the lower the elastic modulus is, the worse the plugging efficiency is. It indicates that the larger the elastic modulus of B-PPG is, the greater the number of particle gel trapped in the core, and the better the plugging efficiency. Moreover, when the elastic modulus is similar, as the particle size increases, the *F_r_* and *F_rr_* increase.

Overall, the B-PPG suspension with lower elastic modulus and larger size, or the B-PPG suspension with higher elastic modulus and lower size, can show good injectivity and plugging efficiency. When the elastic modulus of B-PPG suspension is lower, regardless of particle size, the *F_r_* and *F_rr_* are lower. In this study, when the reservoir permeability is 1.0 μm^2^, and the median diameter (*D*_50_) of particle gel ranges from 525 to 550 μm, the reasonable elastic modulus of B-PPG suspension is in the range of 10.3 to 42.2 Pa. when the median diameter (*D*_50_) of particle gel is 1465 μm, the reasonable elastic modulus of B-PPG suspension cannot exceed 44.1 Pa.

#### 2.4.2. Effect of Matching Coefficient

In order to further clarify the adaptability of B-PPG in different reservoirs, it is necessary to investigate the influence of core permeability on the transport characteristics. The higher the core permeability is, the bigger the pore throat diameter (*D_p_*) is, the lower the matching coefficient is. The matching coefficient was defined as the ratio of median diameter (*D*_50_) to pore throat diameter (*D_p_*). It characterizes the matching relationship between the B-PPG and core permeability, serving as the key factor to ensure successful particle injection into the formation and achieve effective conformance control. Thus, in this section, the #4 B-PPG with an elastic modulus of 42.2 Pa and a *D*_50_ of 525 μm was selected, and the effect of the matching coefficient on the transport characteristics of B-PPG was studied by changing the core permeability. The sand-packs with different permeabilities were filled with quartz sand with different mesh sizes (120–140 mesh, 100–120 mesh, 80–100 mesh, 60–80 mesh). According to the calculation method of matching coefficient *δ* described in Section 4.2.5, the basic parameters of the sand pack used for this experiment are shown in Table 4.

**Figure 7:**
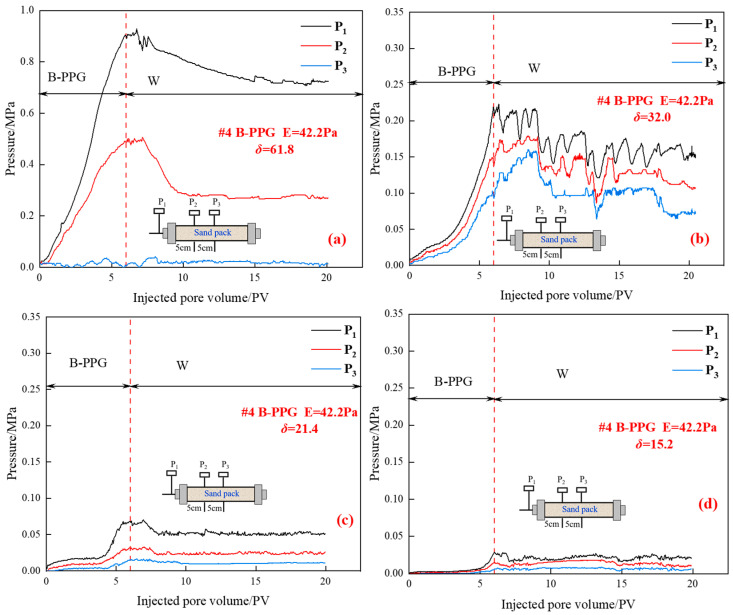
Relationship between the injection pressure and injected pore volumes under different matching coefficients: (**a**) *δ* = 61.8; (**b**) *δ* = 32.0; (**c**) *δ* = 21.4; (**d**) *δ* = 15.2.

Figure 7 shows the relationship between the injection pressure and injected pore volumes during different flooding periods under different matching coefficients.

As shown in Figure 7, during the injection process of B-PPG suspension, when the matching coefficient is 61.8, as the injected pore volume of B-PPG suspension increases, the pressure measuring points P_1_ and P_2_ increase. In contrast, no significant increase in pressure is observed at the pressure measuring point P_3_. When the matching coefficient ranges from 15.2 to 32.0, as the injected pore volume of B-PPG suspension increases, the pressure measuring points P_1_, P_2_, and P_3_ increase. This indicates that the transport ability of B-PPG suspension is improved with the decrease in the matching coefficient. Moreover, with the decrease of the matching coefficient, the maximum injection pressure decreases from 0.91 MPa to 0.029 MPa, which indicates that the injectivity of B-PPG increases. During the subsequent waterflooding, as the pore volume increases, the pressure P1 decreases. When the matching coefficient *δ* is high, the *D*_50_ of B-PPG is much larger than the *D_p_* of the core, and the phenomenon of accumulation and aggregation of B-PPG at the injection end occurs during the process of injecting B-PPG suspension, leading to an increase in pressure. The B-PPG will form plugging at the end face of the core, causing the injection pressure to be higher than the actual value. Moreover, the higher the core permeability, the lower the matching coefficient, and the lower the speed of accumulation and aggregation in the injection end. Thus, the rising speed of injection pressure decreases with the increase of the matching coefficient.

**Figure 8:**
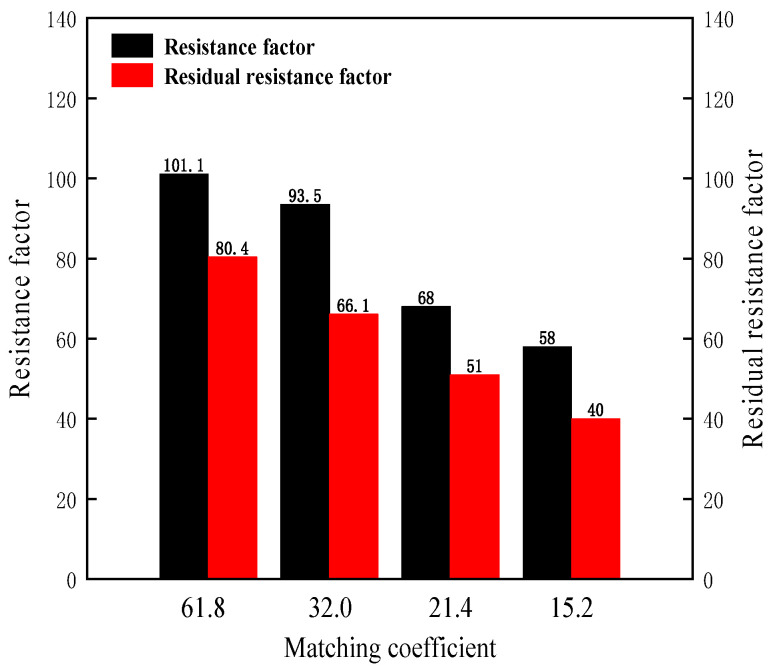
The resistance factors and residual resistance factors versus the matching coefficients.

In order to further illustrate the influence of the matching coefficient on the transport characteristics of B-PPG, the *F_r_* and *F_rr_* were calculated and summarized in Table 5 and Figure 8.

As shown in Figure 8, with the decrease of the matching coefficient *δ*, the resistance factors and residual resistance factors decrease. The higher the core permeability, the lower the matching coefficient, and the lower the speed of accumulation and aggregation in the injection end. The B-PPG can easily enter and transport in the porous media under a low matching coefficient. Thus, the resistance factor decreases with the decrease of the matching coefficient. The residual resistance factor decreases with the decrease of the matching coefficient. Overall, as the core permeability increases from 0.3 μm^2^ to 5.30 μm^2^, the matching coefficient decreases from 61.8 to 15.2. When the matching coefficient is in the range of 15.2 to 32.0, the resistance factor is in the range of 40 to 80.4, which indicates that the B-PPG with an elastic modulus of 42.2 Pa and a *D*_50_ of 525 μm^2^ can form effective plugging.

#### 2.4.3. Effect of Injection Rate

The injection rate of B-PPG suspension will affect the contact time between the B-PPG particle and the pore throat. The lower the injection rate is, the longer the contact time is. With prolonged contact time, the phenomenon of accumulation and aggregation of B-PPG in the injection end is more likely to occur, which will affect the injectivity and migration in porous media. Thus, in this section, the #4 B-PPG with an elastic modulus of 42.2 Pa was selected, and the influence of injection rate was studied. The basic parameters of the sand pack used for experiments are shown in Table 6.

**Figure 9:**
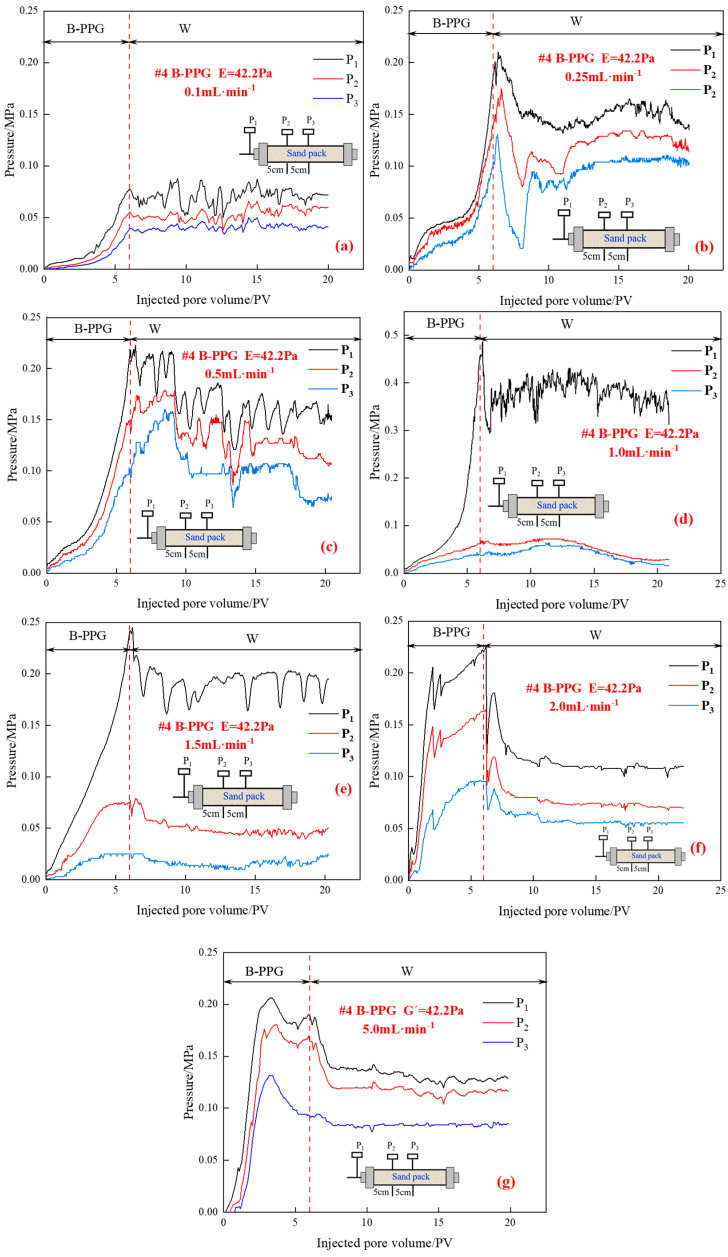
Relationship between the injection pressure and injected pore volumes under different injection rates: (**a**) 0.1 mL·min^−1^; (**b**) 0.25 mL·min^−1^; (**c**) 0.5 mL·min^−1^; (**d**) 1.0 mL·min^−1^; (**e**) 1.5 mL·min^−1^; (**f**) 2.0 mL·min^−1^; (**g**) 5.0 mL·min^−1^.

The relationship between the injection pressure and injected pore volumes during different displacement periods under different injection rates is depicted in Figure 9.

As shown in Figure 9, during the injection process of B-PPG, as the injection rate increases, the rising speed of injection pressure and maximum injection pressure increase first and then decrease. When the injection rate is in the range of 0.25 mL·min^−1^ to 1.0 mL·min^−1^, as the injection rate increases, the external drag force applied to B-PPG particles increases, and the contact time between particle and pore throat decreases, but it is still relatively long. The sedimentation, accumulation, and aggregation of B-PPG particles still easily occur at the injection end, resulting in an increase in the rising speed of injection pressure and maximum injection pressure.

However, when the injection rate ranges from 1.0 mL·min^−1^ to 2.0 mL·min^−1^, the rising speed of injection pressure and maximum injection pressure decreases. When the injection rate is higher than 1.0 mL·min^−1^, on the one hand, the external drag force applied to B-PPG particles increases continuously, and the contact time between particle and pore throat shortens continuously. The B-PPG particles are quickly carried into the sand pack before sedimentation and accumulation. As the injection rate increases, the probability of the phenomena of particle sedimentation and accumulation at the injection end decreases. Moreover, due to the shear effect, the higher the injection rate, the lower the viscosity of the B-PPG suspension and the lower the injection pressure.

**Figure 10:**
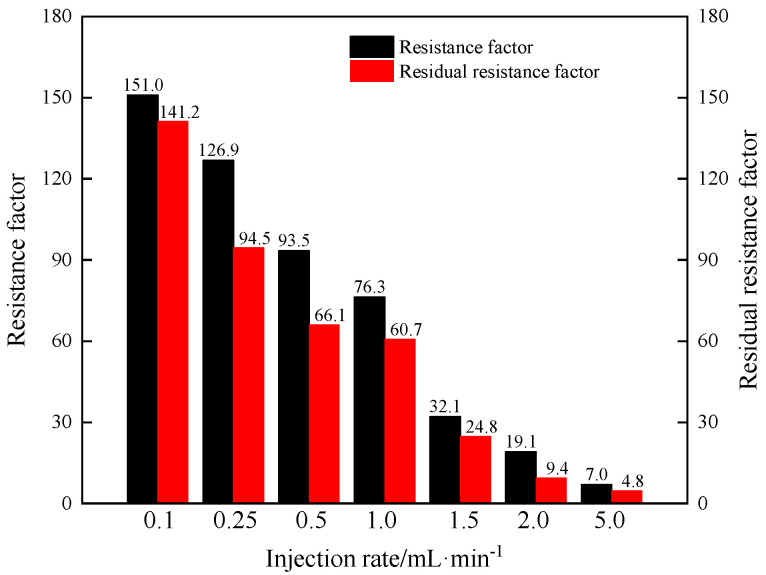
The resistance factors and residual resistance factors versus injection rates.

In order to further describe the transport characteristics, the *F_r_* and *F_rr_* are shown in Table 7 and Figure 10.

As shown in Figure 10, as the injection rate increases, the *F_r_* and *F_rr_* decrease. This indicates that the injectivity of B-PPG suspension increases and plugging efficiency decreases with the increase in injection rate. As the injection rate increases, the probability of the phenomena of particle sedimentation and accumulation at the injection end decreases. Under a high injection rate, the B-PPG particles are quickly injected into the core. Moreover, as the injection rate increases, the viscosity of the B-PPG suspension decreases. The higher the injection rate, the higher the shear force, and the easier it is to break B-PPG particles. Thus, it is easier for particles to be injected into the core, and the injectivity of the B-PPG suspension increases. The easier it is to break B-PPG particles, the worse the plugging efficiency is. Overall, to achieve good injectivity and plugging efficiency of B-PPG suspension, the injection rate should be in the range of 0.5 mL·min^−1^ to 1.5 mL·min^−1^.

In previous studies, when the permeability was in the range of 1.6 to 3.2 μm^2^, B-PPG with the volume/size swelling factor of 47.3/3.6 and a median diameter of 430 μm could migrate to the deep reservoir, significantly improving the reservoir heterogeneity. When the injection rate was low, B-PPG with the volume swelling factor of 40 and an elastic modulus of 70 Pa was suitable for fractured reservoirs with a permeability exceeding 0.1 × 10^−3^ μm^2^. The B-PPG with an elastic modulus of 0.1~1 Pa and a median diameter of 607.3 μm exhibited limited injectivity in the 1526 × 10^−3^ μm^2^ and had good properties of injection in the 8353 × 10^−3^ μm^2^. The above study focuses on the influence of injection rate and permeability on the transport characteristics of B-PPG in porous media, while the influence of particle mechanical properties on their transport characteristics remains poorly understood. Thus, the elastic modulus and matching coefficient are introduced to clarify the transport characteristic of B-PPG. In addition, due to the injection rate affecting the contact time between the particle and the pore throat, the injection rate was studied. The results show that when the reservoir permeability is 1.0 μm^2^, and the *D*_50_ of particle gel ranges from 525 to 550 μm, the reasonable elastic modulus of B-PPG ranges from 10.3 to 42.2 Pa. When the median diameter (*D*_50_) of particle gel is 1465 μm, the reasonable elastic modulus of B-PPG suspension should not exceed 44.1 Pa. The core permeability increases from 0.3 μm^2^ to 5.30 μm^2^; the matching coefficient decreases from 61.8 to 15.2. When the matching coefficient is in the range of 15.2 to 32.0, the residual resistance factor is in the range of 40 to 80.4, which indicates that the B-PPG with an elastic modulus of 42.2 Pa and a *D*_50_ of 525 μm can be injected and form effective plugging. To achieve good injectivity and plugging efficiency of B-PPG suspension, the injection rate should be in the range of 0.5 mL·min^−1^ to 1.5 mL·min^−1^.

## 3. Conclusions

In this study, the influence of elastic modulus, matching coefficient, and injection rate on the transport characteristics of B-PPG in porous media was investigated. Some remarking conclusions can be made.

(1) When the particle size of B-PPG is similar, as the elastic modulus increases, the *F_r_* and *F_rr_* increase. When the elastic modulus is similar, with the increase of the particle size, the *F_r_* and *F_rr_* increase. When the reservoir permeability is 1.0 μm^2^, and the *D*_50_ of particle gel ranges from 525 to 550 μm, the reasonable elastic modulus of B-PPG ranges from 10.3 to 42.2 Pa. When the median diameter (*D*_50_) of particle gel is 1465 μm, the reasonable elastic modulus of B-PPG suspension should not exceed 44.1 Pa.

(2) With the decrease of the matching coefficient, the *F_r_* and *F_rr_* decrease, reflecting the improvement of injectivity and the weakening of plugging efficiency. The higher the core permeability, the lower the matching coefficient. When the reservoir permeability is in the range of 0.30 to 5.30 μm^2^, the residual resistance factor is in the range of 40 to 80.4, which indicates that the B-PPG with an elastic modulus of 42.2 Pa and a *D*_50_ of 525 μm can form effective plugging.

(3) As the injection rate increases, the *F_r_* and *F_rr_* decrease, reflecting the improvement of injectivity and the weakening of plugging efficiency. Therefore, to achieve good injectivity and plugging efficiency of B-PPG suspension, the injection rate should be in the range of 0.5 mL·min^−1^ to 1.5 mL·min^−1^.

## 4. Materials and Methods

### 4.1. Experimental Materials

**Figure 11:**
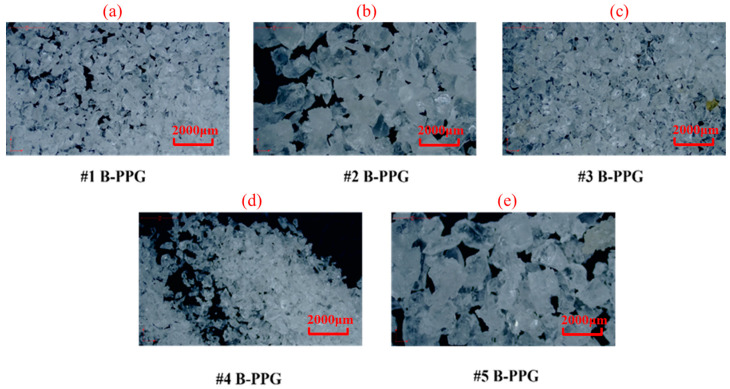
The appearance of five branched-preformed particle gels (B-PPGs) with different elastic moduli before swelling: (**a**) #1; (**b**) #2; (**c**) #3; (**d**) #4; (**e**) #5.

Five kinds of branched-preformed particle gel with different elastic moduli were provided by the Research Institute of Exploration and Development of Shengli Oilfield (China). The appearance of five B-PPGs before swelling is shown in Figure 11. From an appearance perspective, the five B-PPGs have no difference except for the particle size, and all have a white color. The five B-PPGs were numbered #1, #2, #3, #4, and #5. According to the elastic property evaluation standard of Shengli oilfield, the elastic modulus (G′) for swollen B-PPG particles in brine was measured by conducting rheological measurement experiments on a HAAKE MARS 40 (Thermo Fisher Scientific (China) Co., Ltd. (Shanghai, China)) rotational rheometer as follows: (1) The linear viscoelastic region was determined and the stress was set to 0.1 Pa, the frequency was set to 1.0 Hz, and the plate spacing was set to 0.2 mm. (2) Then the B-PPG suspension was placed on a plate at a constant temperature of 30 °C for 3 min. The elastic modulus of each B-PPG sample was tested 3 times to ensure that the error between each measured value and the arithmetic mean did not exceed 10%. The elastic modulus of swollen B-PPG samples is shown in Table 8.

The calcium chloride (CaCl_2_), sodium chloride (NaCl), and magnesium chloride (MgCl_2_) were purchased from Aladdin Reagent. The synthetic formation brine was prepared using agents and distilled water. The composition of the brine solution with total dissolved solids (TDS) of 21,190.35 mg·L^−1^ is shown in Table 9.

### 4.2. Experimental Methods

#### 4.2.1. Measurement of Swelling Ratio

The swelling ratio is an important parameter for evaluating the properties of B-PPG. The swelling ratio of B-PPG can be measured by comparing the mass of the particles before and after swelling [47]. To evaluate the swelling ratio, a certain mass of B-PPG dry powder was added to the formation brine at 30 °C, and then the swollen B-PPG suspension with a concentration of 800 mg·L^−1^ was prepared by stirring for 2.0 h. Then the swollen particles in the suspension were filtered, and the mass of the swollen particles was weighed. The swelling ratio can be calculated by Equation (1) as follows:

(1) $$SR=\frac{m_{1}-m_{0}}{m_{0}}$$

where the *SR* is the swelling ratio of B-PPG; *m*_0_ is the mass of dry powder; *m*_1_ is the mass of B-PPG after swelling.

#### 4.2.2. Measurement of Particle Size Distribution Characteristics

The particle size distribution of B-PPG after swelling was determined using a Bettersize2600 (Bettersize Instruments (China) Ltd., Dandong, China) laser particle size analyzer as follows: (1) The B-PPG suspension of 800 mg·L^−1^ was obtained by adding the determined amount of dry powder into synthetic formation brine and stirring at 300 r·min^−1^ for 2.0 h. (2) Then, the particle size distribution characteristics and the median diameter (*D*_50_) of B-PPG after swelling were measured by the laser particle size analyzer.

#### 4.2.3. Fourier Transform Infrared Characterization

The B-PPG was prepared using the KBr pressing method to characterize the molecular structure. A small amount of the B-PPG sample was mixed with dry KBr powder and compressed under high pressure to form a thin layer. The experimental instrument was a Tensor 27 Fourier transform infrared spectrometer (Bruker, Germany). FTIR measurements were performed using the Tensor 27.

#### 4.2.4. Evaluation of Transport Characteristics of B-PPG

**Figure 12:**
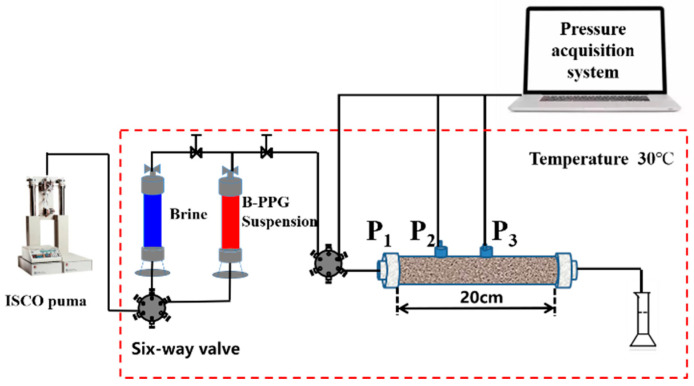
Schematic diagram of the sand-pack flooding experimental apparatus.

The transport characteristics of B-PPG in porous media were evaluated by performing core flow experiments. A sand pack (Φ2.5 cm × 20 cm) was used as the core, with pressure measuring points (P_1_, P_2_, and P_3_) located at the injection end, 5 cm, and 10 cm along the sand pack. Figure 12 shows the schematic of the core flow experimental apparatus. All the experiments were performed below the ambient temperature (30 °C). The experimental processes are listed below: (1) The sand-packs with different permeabilities were prepared using the wet-packing method. During the filling process, quartz sand and simulated formation brine were alternately added and compacted. The sand-pack pore volume was determined as the volume of the used simulation formation brine, and the porosity was calculated. (2) Brine flooding was performed by injecting the synthetic formation brine at a certain flow rate until the injection pressure was stable. The stable injection pressure ∆*P_wa_* was recorded, and the permeability was determined. (3) About 6.0 PV B-PPG suspension was injected, and subsequent brine flooding was performed until the total injected pore volume reached 20.0 PV. The injection pressures ∆*P_B-PPG_* and ∆*P_wb_* during B-PPG suspension injection and the subsequent water flooding process were recorded. The injection rates during B-PPG suspension injection and the subsequent water flooding process were the same.

The transport characteristics of B-PPG in porous media were evaluated by analyzing the injectivity and plugging efficiency. The injectivity and plugging efficiency are characterized by the resistance factor (*F_r_*) and the residual resistance factor (*F_rr_*), which can be calculated according to the following equations:

(2) $$Fr=\frac{\Delta P_{B-\mathrm{PPG}}}{\Delta P_{wa}}$$

(3) $$Frr=\frac{\Delta P_{wb}}{\Delta P_{wa}}$$

The *F_r_* and *F_rr_* were calculated according to the injection pressure when the injection volume reached 6.0 PV and 20.0 PV, with the injection rates during the B-PPG suspension injection and the subsequent water flooding process being the same.

The injectivity (*I*) of B-PPG was defined as the ratio of injection rate (*Q*) to injection pressure ∆*P_B-PPG_*, as described by Equation (4). A higher value of *I* means that injection is easier. The plugging efficiency (*η*) was calculated using Equation (5).

(4) $$I=\frac{Q}{\Delta P_{B-\mathrm{PPG}}}$$

(5) $$\eta =(1-\frac{1}{F_{rr}})\times 100\%$$

#### 4.2.5. Calculation Method of Matching Coefficient Between B-PPG and Core Pore Throat

The matching relationship between the particle size of B-PPG and the pore throat diameter was characterized by the matching coefficient, which is an important factor affecting its injectivity and plugging capacity. The matching coefficient *δ* was defined as the ratio of median diameter (*D*_50_) to pore throat diameter (*D_p_*). The *D*_50_ of B-PPG was measured by a laser particle size analyzer, and the pore throat diameter of porous media was determined by the Kozeny–Carman equation [39,40]:

(6) $$Dp=\sqrt{\frac{16f_{ck}\tau^{2}K}{\varphi }}$$

where the *f_ck_τ*^2^ is a constant, and its numerical value is related to the complexity of the pore structure of porous media: 4.5 ≤ *f_ck_τ*^2^ ≤ 5.1. Since the pore structure of the sand pack core used in these experiments was relatively simple, the value of *f_ck_τ*^2^ was set at 4.5.

## Figures and Tables

**Table 1:** *D*_50_ values of B-PPG suspensions with different elastic moduli.

B-PPG	Elastic Modulus/Pa	Particle Size		
		*D*_10_/μm	*D*_50_/μm	*D*_90_/μm
#1	0.7	259	550	889
#2	3.4	992	1465	1898
#3	10.3	228	542	942
#4	42.2	247	525	917
#5	44.1	1013	1466	1856

**Table 2:** Sand pack parameters of the displacement experiment with different elastic moduli.

No.	B-PPG	Elastic Modulus /Pa	Particle Size *D*_50_/μm	Injection Rate /mL·min^−1^	Permeability /μm^2^	Porosity /%
1	#1	0.7	550	0.5	1.20	30.8
2	#2	3.4	1465		1.20	31.1
3	#3	10.3	542		1.00	30.6
4	#4	42.2	525		1.18	31.6
5	#5	44.1	1466		1.08	30.4

**Table 3:** The resistance factors and residual resistance factors versus elastic moduli.

No.	Elastic Modulus /Pa	*D*_50_ /μm	Permeability /μm^2^	∆*P_wa_* /MPa	∆*P_B-PPG_* /MPa	∆*P*_wb_ /MPa	*F*_r_	*F*_rr_
1	0.7	550	1.20	0.0023	0.042	0.037	18.3	16.1
2	3.4	1465	1.20	0.0023	0.113	0.057	49.1	24.8
3	10.3	542	1.00	0.0030	0.188	0.146	62.7	48.7
4	42.2	525	1.18	0.0024	0.215	0.152	93.5	66.1
5	44.1	1466	1.08	0.0025	0.276	0.268	110.4	107.2

**Table 4:** Sand pack parameters of the displacement experiment with different matching coefficients.

No.	Elastic Modulus /Pa	*D*_50_ /μm	Injection Rate /mL·min^−1^	Permeability /μm^2^	Porosity /%	*D_p_* /μm	Matching Coefficient *δ*
6	42.2 Pa	525	0.5	0.30	30.2	8.5	61.8
7				1.18	31.6	16.4	32.0
8				2.70	32.4	24.5	21.4
9				5.30	32.1	34.5	15.2

**Table 5:** The resistance factors and residual resistance factors versus the matching coefficients.

No.	*D*_50_ /μm	Permeability /μm^2^	*D_p_* /μm	*δ*	∆*P*_wa_ /MPa	∆*P*_B-PPG_ /MPa	∆*P*_wb_ /MPa	*F*_r_	*F*_rr_
6	525	0.30	8.5	61.8	0.0090	0.910	0.724	101.1	80.4
7		1.18	16.4	32.0	0.0024	0.215	0.152	93.5	66.1
8		2.70	24.5	21.4	0.0010	0.068	0.051	68.0	51.0
9		5.30	34.5	15.2	0.0005	0.029	0.020	58.0	40.0

**Table 6:** The basic properties of the sand pack used for the flooding experiment under different injection rates.

No.	*D*_50_ /μm	Elastic Modulus /Pa	Injection Rate /mL·min^−1^	Permeability /μm^2^	Porosity /%
10	525	42.2	0.25	0.95	31.2
11			0.50	1.18	31.6
12			1.00	0.92	30.8
13			1.50	1.07	32.1
14			2.00	0.95	31.0

**Table 7:** The resistance factors and residual resistance factors versus injection rates.

No.	Injection Rate /mL·min^−1^	Permeability /μm^2^	∆*P*_wa_ /MPa	∆*P*_B-PPG_ /MPa	∆*P*_wb_ /MPa	*F*_r_	*F*_rr_
10	0.10	1.08	0.00051	0.077	0.072	151.0	141.2
11	0.25	0.95	0.00145	0.184	0.137	126.9	94.5
12	0.50	1.18	0.00240	0.215	0.152	93.5	66.1
13	1.00	0.92	0.00590	0.450	0.358	76.3	60.7
14	1.50	1.07	0.00750	0.241	0.186	32.1	24.8
15	2.00	0.95	0.01150	0.220	0.108	19.1	9.4
16	5.00	1.01	0.02700	0.190	0.129	7.0	4.8

**Table 8:** The elastic moduli of B-PPGs.

No.	#1	#2	#3	#4	#5
Elastic modulus/Pa	0.7	3.4	10.3	42.2	44.1

**Table 9:** Composition of synthetic formation brine.

Type of Ions	Na^+^	Ca^2+^	Mg^2+^	Cl^−^
Concentration of ions/(mg·L^−1^)	7466.15	428	255.7	13,040.5

## Data Availability

Data are available upon request from the authors.
